# Comparative performance of sponge versus flocked swabs for culture-based and metagenomic detection of microbial contamination in the healthcare environment

**DOI:** 10.1017/ice.2025.87

**Published:** 2025-08

**Authors:** Matthew J. Ziegler, Sean Loughrey, Selamawit Bekele, Elizabeth Huang, Pam Tolomeo, Michael Z. David, Ebbing Lautenbach, Laurel J. Glaser, Brendan J. Kelly

**Affiliations:** 1 Division of Infectious Diseases, Department of Medicine, Perelman School of Medicine, University of Pennsylvania, Philadelphia, PA, USA; 2 Department of Biostatistics, Epidemiology, and Informatics, Perelman School of Medicine, University of Pennsylvania, Philadelphia, PA, USA; 3 Center for Clinical Epidemiology and Biostatistics, Perelman School of Medicine, University of Pennsylvania, Philadelphia, PA, USA; 4 Clinical Pathology and Laboratory Medicine, Perelman School of Medicine, University of Pennsylvania, Philadelphia, PA, USA

## Abstract

**Background:**

Identifying optimal methods for sampling surfaces in the healthcare environment is critical for future research requiring the identification of multidrug-resistant organisms (MDROs) on surfaces.

**Methods:**

We compared 2 swabbing methods, use of a flocked swab versus a sponge-stick, for recovery of MDROs by both culture and recovery of bacterial DNA via quantitative 16S polymerase chain reaction (PCR). This comparison was conducted by assessing swab performance in a longitudinal survey of MDRO contamination in hospital rooms. Additionally, a laboratory-prepared surface was also used to compare the recovery of each swab type with a matching surface area.

**Results:**

Sponge-sticks were superior to flocked swabs for culture-based recovery of MDROs, with a sensitivity of 80% compared to 58%. Similarly, sponge-sticks demonstrated greater recovery of *Staphylococcus aureus* from laboratory-prepared surfaces, although the performance of flocked swabs improved when premoistened. In contrast, recovery of bacterial DNA via quantitative 16S PCR was greater with flocked swabs by an average of 3 log copies per specimen.

**Conclusions:**

The optimal swabbing method of environmental surfaces differs by method of analysis. Sponge-sticks were superior to flocked swabs for culture-based detection of bacteria but inferior for recovery of bacterial DNA.

## Introduction

Surface contamination with multidrug-resistant organisms (MDROs) in the healthcare environment is a reservoir for the acquisition, colonization, and infection of patients.^1^ Accordingly, research on the effectiveness of environmental cleaning and the role of surfaces in the transmission of pathogens is increasingly important. However, methods for sampling of environmental surfaces are not standardized, and differences in sensitivity across methods can lead to inconsistent conclusions.

Prior research has relied on several methods for environmental bacterial sampling, including RODAC plates, sponge-sticks, and cotton or flocked swabs.^2–6^ In contrast, surface sampling for metagenomic analysis has relied mostly on flocked swabs.^7–9^ Minimal research has been conducted to evaluate the comparative performance of sampling strategies of surfaces in the healthcare environment,^10^ and no prior study has compared the effectiveness of 2 common methods of surface sampling for both culture-based recovery and molecular analysis. To inform sampling methods, we compared 2 environmental sampling methods (ie, sponge vs flocked swabs) to determine the differential recovery by culture-based and molecular techniques for the assessment of bacterial contamination in the healthcare environment.

## Materials and methods

### Study design, setting, and population

We conducted comparative swabbing in hospital rooms as part of a study of environmental surface contamination in intensive care units (ICU).^11^ We enrolled hospital rooms of newly admitted patients who had a positive clinical or surveillance culture for an eligible MDRO within the prior 30 days. Eligible MDROs included methicillin-resistant *Staphylococcus aureus* (MRSA), vancomycin-resistant Enterococci (VRE), *Clostridioides difficile* (CDIFF), extended-spectrum beta-lactamase-producing Enterobacterales (ESBL), and carbapenem-resistant Enterobacterales (CRE). Sampling occurred longitudinally until discharge from the enrolling ICU room or day 28. Approval was granted by the University of Pennsylvania Institutional Review Board (IRB protocol #833724).

### Specimen collection

**Figure 1.**
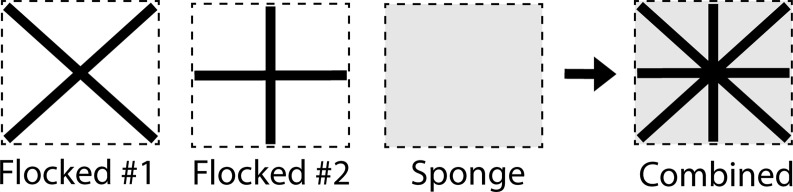
Specimen collection approach. Each section demonstrates the pattern applied when swabbing was performed. The surface area sampled (dashed line) represents approximately 50 in^2^.

Multiple surfaces were sampled with each swab, with composite sampling groups including (1) *near-patient*, comprising the patient bed railing, the television remote control, the nurse call button, and the overbed tray table; (2) *intermediate distance*, comprising the intravenous fluid pump control pad, the supply cart keypad, the in-room computer keyboard, and mouse; and (3) *remote surfaces*, distant from the patient and in close proximity to wastewater surfaces, comprising the light switch, doorknob, toilet handle and seat, and the handles and basin of the bath and/or in-room sinks. Sampling composite groups was chosen to approximate 350 in^2^ for all surfaces combined, with an average surface area of any individual surface within a composite zone being 50 in^2^ (ie, large flat surfaces were sampled at the maximum surface area, while smaller surfaces such as a nurse call button were not). Research staff were trained to identify the boundaries of sampling areas using landmarks present on each surface. To repeatedly sample the same surface with multiple methods, we first used 2 flocked swabs, in an “x” and “+” pattern, respectively, within the bounds of the area to be sampled, followed by sampling of the entire surface area within the bounds with the sponge-stick (Figure 1). This resulted in a smaller surface area sampled by each individual flocked swab (Table 1) but allowed repeated sampling of the same boundaries selected for individual surfaces. The sponge-stick (3M sponge-stick with neutralizing buffer) underwent processing for both 16S qPCR and bacterial culture, while the first flocked swab (Copan FLOQ swab) was reserved for 16S qPCR, and the second flocked swab (BD ESwab with Liquid Amies media) was reserved for bacterial culture. Flocked swabs used for environmental sampling within patient rooms were applied to surfaces without premoistening.

**Table 1.** Overview of swab characteristics and processing

Swab	Material	Size	Premoistened	Average area per surface^a^	Dilution	Homogenization	Final volume
3M sponge-stick with neutralizing buffer (3M SSL10NB)	Cellulose	1.5 × 3 in	Yes, 10 mL	50 in^2^	45 mL PBST	Yes^b^	5 mL
BD ESwab w/ Liquid Amies (BD 220245)	Nylon	1 in	No	20 in^c^	No	Yes^d^	1 mL
Copan Floq Swab Regular Tip (COPAN 502C)	Nylon	1 in	No	20 in^c^	No	No	N/A

Note. PBST, phosphate-buffered saline.

a Multiple surfaces of varying size were sampled per specimen. Sampling was standardized to achieve matching surface areas between sampling events and patient rooms.

b Seward 400C Stomacher for 1 minute at 200 RPM. After concentration via centrifugation, the final pellet was resuspended by vortexing.

c Swabs were applied in a linear pass. The swab tips measured approximately 1 inch in length.

d Vortexing performed with swab submerged in Liquid Amies media.

### MDRO culture

Sponge-stick specimens for bacterial culture were collected in stomacher bags to which phosphate-buffered saline with 0.02% Tween (PBST) was added, with mixture using a mechanical homogenizer (Seward). After centrifugation, 50 μL of the cell pellet was plated on each of 6 selective media, including BD MRSA CHROMagar, HardyCHROM CRE, Hardy CHROMagar KPC, HardyCHROM ESBL, Thermo Scientific SPECTRA VRE, and Anaerobe Systems CCFA-HT. Flocked swab specimens were processed by placing 50 μL of BD ESwab Amies transport media into each of the 6 selective media. Cultures were incubated aerobically for 18–24 hours at 35–37 °C, with the exception of Anaerobe Systems CCFA-HT, which was incubated in anaerobic conditions for 48–72 hours. All bacterial colonies with appropriate morphology and chromogenic features were subcultured to blood agar and confirmed by MALDI-TOF, with the exception of MRSA, which were interpreted as true positives based on selective chromogenic culture alone.

### Culture recovery from laboratory-prepared surfaces

To compare the culture recovery of a flocked swab to a sponge-stick using the same surface area, we prepared a standard concentration of bacteria on a polystyrene surface in a biosafety cabinet. Using *Staphylococcus aureus* (SA) strain 493NT, an initial inoculum was created by McFarland optical density (0.5 McFarland). Serial dilutions and varying inoculation amounts were applied to surfaces and control plates to achieve a readable number of colonies to be present on a blood agar plate. Additional details of surface preparation and sampling are present in the Supplemental Methods. Solution concentrations were determined by colony count of control plates. The average amount of CFU/mL from colony counts was compared to the control concentrations. The experiment above was then repeated in a similar fashion to compare the recovery of a flocked swab without premoistening, to a flocked swab that was premoistened by dipping the swab into liquid Amies media and removing excess fluid by ringing the swab against the inside of the container.

### 16S qPCR

Flocked swabs for 16S qPCR were frozen at −80 °C prior to DNA extraction and sequencing. Sponge-sticks were processed as described above (see MDRO culture) with 300 μL of the cell pellet reserved for DNA extraction. DNA extraction was performed using the MoBio PowerSoil DNA isolation kit (Qiagen, Hilden, Germany). The 16S rRNA gene quantitative polymerase chain reaction PCR (qPCR) was performed targeting the V1–V2 hypervariable region, with primers BSF8 (5’-AGAGTTTGATCCTGGCTCAG-3’) and BSR357 (5’-CTGCT GCCTYCCGTA-3’) and probe 5’-TAACAC ATGCAAGTCGA-3’. The standard curve was generated with a plasmid containing the 16S rRNA gene derived from *Streptococcus*.

### Statistical analysis

The primary outcome for each experiment was the differential recovery of bacteria or bacterial DNA from sponge versus flocked swabs of surfaces. To evaluate differences in the sensitivity of swab type for recovery of MDROs within patient rooms, we describe the sensitivity of each medium for detection of an MDRO by comparing matched pairs of swabs. A positive culture by either swab type was determined to be a true positive. In addition, we performed a Bayesian mixed-effects binomial regression, examining the difference in sensitivity for each media type. Swab comparisons were visualized via posterior distributions, and comparisons were made using posterior contrasts (and 95% posterior credible intervals). To compare the recovery of bacteria from laboratory-controlled experimental prepared surfaces, we used descriptive statistics. The outcome for the recovery of bacterial DNA via 16S quantitative PCR was the log-transformed copies of the 16S rRNA gene sequence modeled using generalized linear regression using a random intercept for surface type nested within patient room.

## Results

### Culture-based detection of MDROs

**Figure 2.**
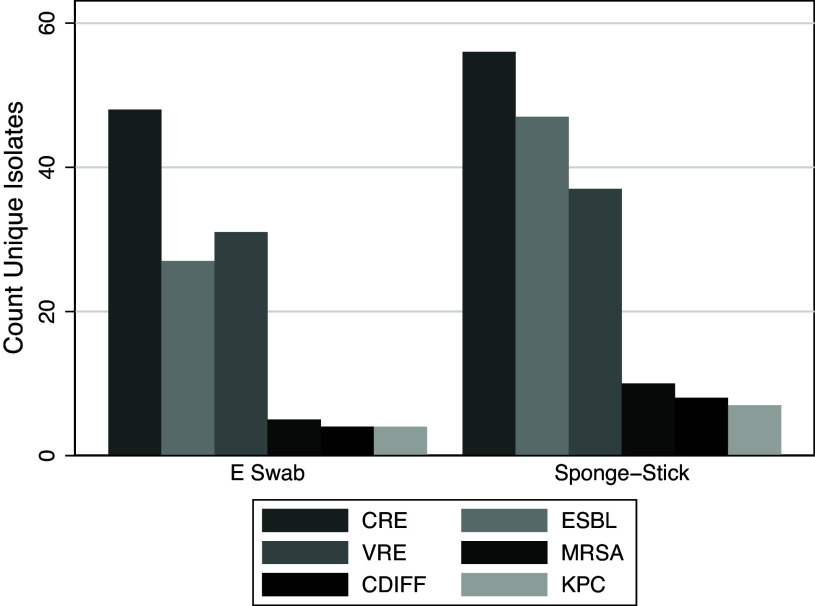
Count of unique bacterial isolates recovered by selective media. *Note.* Unique bacterial isolates by genus, species, and resistance phenotype. “CDIFF,” *Clostridioides difficile*; “CRE,” carbapenem-resistant Enterobacterales; “ESBL,” extended-spectrum beta-lactamase-producing Enterobacterales; “KPC,” *Klebsiella pneumoniae* carbapenemase-producing Enterobacterales; “MRSA”, methicillin-resistant *Staphylococcus aureus*; “VRE,” vancomycin-resistant Enterococci.

**Figure 3.**
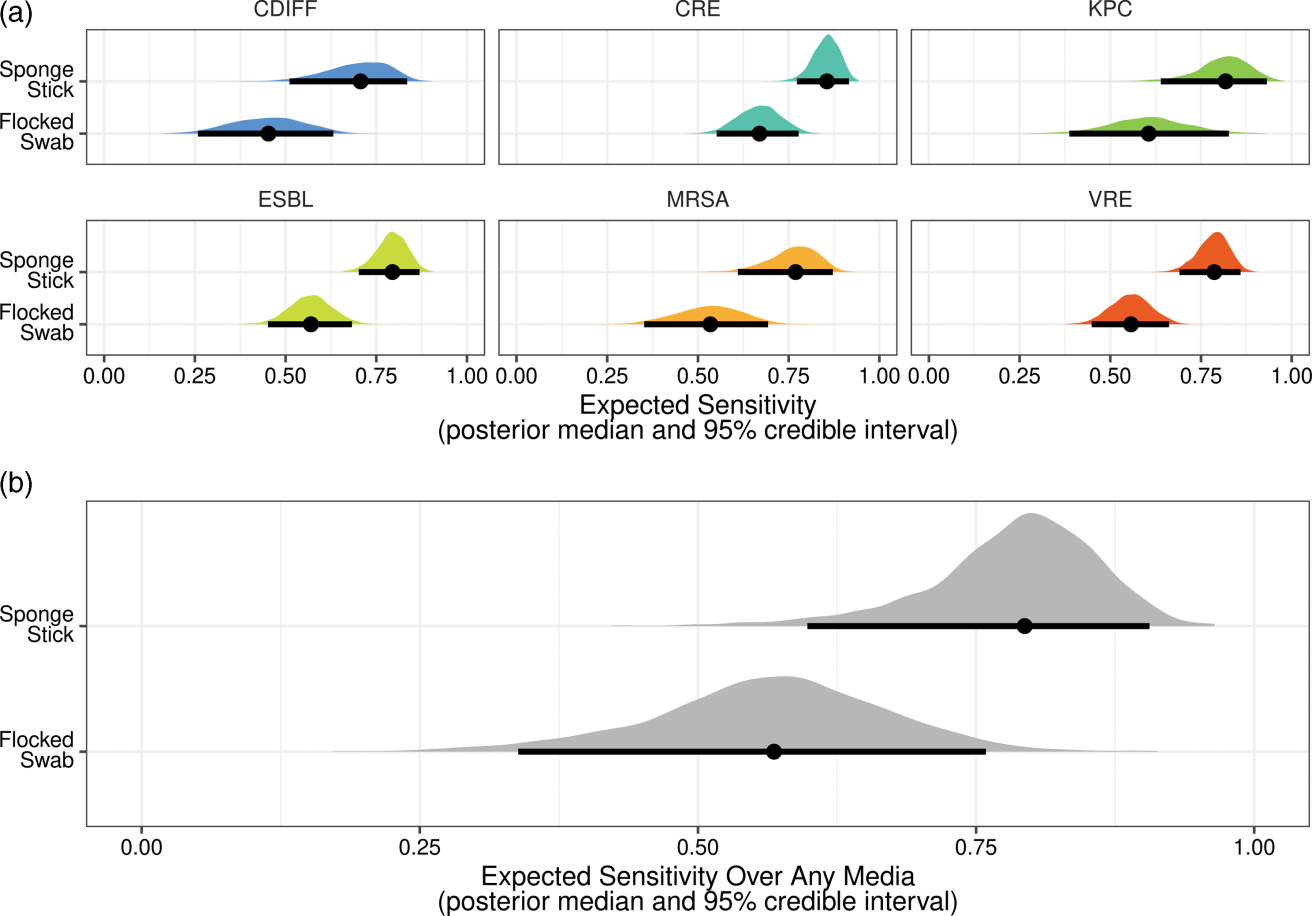
Sensitivity for multidrug-resistant organisms detected by different media with sponge-stick versus flocked swab sampling, from a Bayesian mixed-effects regression model with a random effect for media type. The point represents the best estimate (posterior median) of the sensitivity, and the black lines represent the 95% posterior credible interval. Densities show the full posterior distribution of expected sensitivity, colored by media type (A) and shown in gray for the pooled effect over all media (B).

A total of 966 swabs were included in this analysis (483 Flocked swabs and 483 sponge-sticks). These swabs were obtained from 61 patient rooms over 161 total visits. Each swab was plated to multiple selective media in parallel, resulting in 4,920 total cultures. We recovered a total of 284 target MDROs from in-room cultures, 165 using the sponge-stick method and 119 using the Flocked swab method. Bacterial recovery by media type is displayed in Figure 2. Using a matched pair analysis, the sponge-stick method demonstrated superior sensitivity across all media types, with an aggregate sensitivity of 80% with the sponge-stick compared to 58% with the flocked swab (Table 2). To further define differences in recovery across different culture media, we examined the posterior distribution of a Bayesian mixed-effects regression model with a random effect for the media type. We found that sponge-stick sampling demonstrated consistently higher sensitivity across all tested selective media for MDROs (Figure 3). For *Clostridioides difficile*, the sponge-stick was 24.6% (95% CrI, 13.5%–35.8%) more sensitive than the flocked swab; for CRE, 18.3% (95% CrI, 9.9%–27.8%) more sensitive; for KPC, 20.5% (95% CrI, 9%–32.8%) more sensitive; for ESBL, 22.3% (95% CrI, 12.3%–32.2%) more sensitive; for MRSA, 23.1% (95% CrI, 12.5%–34.1%) more sensitive; and for VRE, 22.7% (95% CrI, 12.6%–32.6%) more sensitive. In aggregate, the sponge-stick was found to be 21.8% (95% CrI, 10.9%–33.5%) more sensitive than a flocked swab for MDRO detection.

**Table 2.** Sensitivity of swabbing method for culture-based recovery of multidrug-resistant organisms by different media

Media	Sensitivity^a^ (%) sponge-stick	Sensitivity^a^ (%) flocked swab	Surface samples (N)	Difference in sensitivity
CDIFF	67	33	468	34
CRE	84	73	483	11
ESBL	84	52	483	32
KPC	100	50	60	50
MRSA	83	42	483	41
VRE	73	61	483	12
Aggregate	80	58	2460	22

a Sensitivity based on composite reference standard. Positive by either swabbing method assumed to be a true positive.

Note. CDIFF, *Clostridioides difficile*; CRE, carbapenem-resistant Enterobacterales; ESBL, extended-spectrum beta-lactamase-producing Enterobacterales; KPC, *Klebsiella pneumoniae* carbapenemase-producing Enterobacterales; MRSA, methicillin-resistant *Staphylococcus aureus*; VRE, vancomycin-resistant Enterococci.

### Validation of differential culture-based recovery from laboratory-prepared surfaces

To validate our real-world findings, we compared recovery by bacterial culture from laboratory-prepared surfaces sampled by either sponge-sticks or flocked swabs. In this validation experiment, the sponge-stick recovery was greater than recovery using a flocked swab in both low and high concentrations and from both dry and wet surfaces. Recovery from wet surfaces was an average of 59.40% with the sponge-stick compared to 7.95% using a flocked swab across concentrations (Supplementary Table 1). Recovery from dry surfaces was much lower, an average of 1.43% using a sponge-stick compared to 0.37% using a flocked swab. When repeated to directly compare flocked swab recovery using either a dry or premoistened swab, recovery was increased using a premoistened swab (33.86% vs 13.05% from wet surfaces and 1.37% vs 0.07% from dry surfaces), averaged across low and high concentration surfaces (Supplementary Table 2).

### Recovery of bacterial DNA

**Figure 4.**
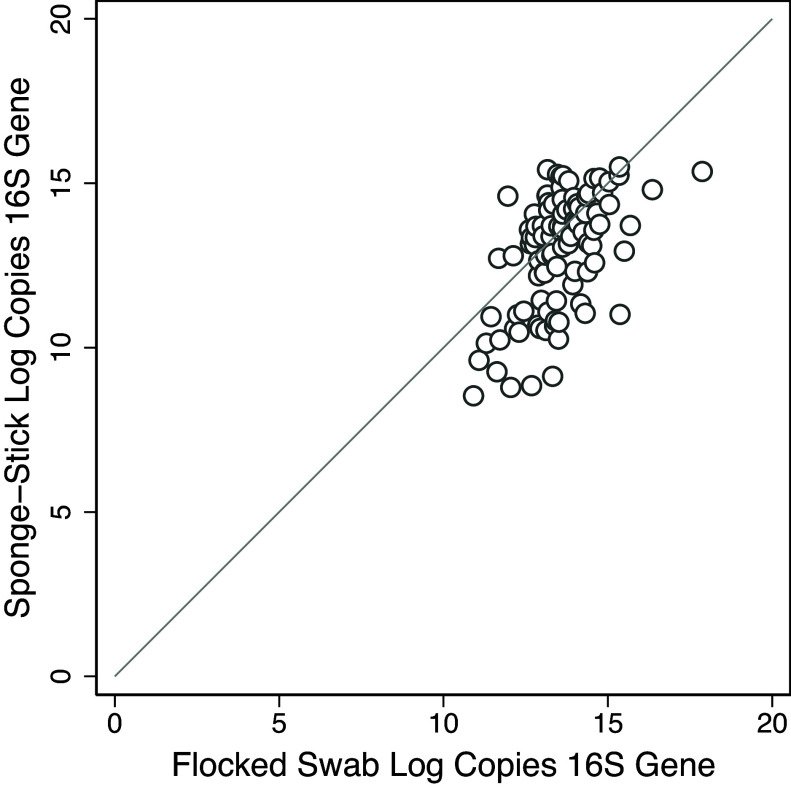
Scatterplot of bacterial 16S rRNA gene sequences detected by quantitative PCR by swabbing method. Each circle represents a paired sample of hospital surfaces. Circles below the solid line (y = x) represent specimens where the flocked swab yielded fewer copies of the 16S bacterial gene.

A total of 246 specimens from patient rooms underwent DNA extraction with sequencing of the bacterial 16S rRNA gene sequence (123 flocked swabs and 123 sponge-sticks). The sponge-sticks had a median of 12.8 log copies of the 16S rRNA gene sequence (IQR, 10.1–14.0) compared to a median of 13.4 (IQR, 12.8–14.0) with the flocked swab. There were a total of 24 (19.5%) sponge specimens where no bacterial DNA was detected compared to 0 with the flocked swab. Of these 24 specimens where no bacterial DNA was recovered from sponge-sticks, there was no apparent unifying factor. Sponge-stick failures were distributed among 12 patient rooms on 3 hospital units and across all surface types (9 [38%] from near-patient area, 10 [42%] from distant surfaces, and 5 [21%] from bathroom surfaces). Excluding collection episodes where DNA was not detected with the sponge-stick method, the sponge-sticks had a median of 13.3 log copies of 16S rRNA gene sequence (IQR, 11.4–14.3) compared to a median of 13.4 (IQR, 12.8–14.1) with flocked swabs. 16S qPCR results by swab type are displayed in Figure 4. Using mixed-effects generalized linear regression with a random intercept for patient and surface, we found that flocked swabs were associated with an increase of an average of 3.0 log copies per specimen in comparison to the sponge-stick method (*β* 3.0 log copies, [95% CI, 2.1–3.9], *P* <.001).

## Discussion

In this project, we identified that the sponge-stick modality is superior to flocked swab collection for culture-based detection of multidrug-resistant bacteria and inferior for PCR detection of the bacterial 16S rRNA gene sequence. In contrast, flocked swabs performed significantly better for the recovery of bacterial DNA from the environment. The decreased culture-based recovery of the flocked swab modality may be due to many factors. First, while the flocked design increases the surface area of the swab and improves elution, the sponge-stick is larger in size and may allow greater total collection of debris. Secondly, the sponge-stick is premoistened while the flocked swabs utilized for patient room collection were not. We had initially hypothesized that premoistened flocked swabs would result in poorer collection due to lost static charge and potential loss of surface bacteria through the spread of droplets rather than adhesion to the swab. Additionally, studies comparing dry versus premoistened swabs for MRSA nares collection did not show a benefit from premoistened swabs, even in artificial models.^12–14^ However, when we directly compared dry versus premoistened flocked swabs using laboratory-prepared surfaces, the premoistened swab performed better on both wet and dry surfaces. A similar finding was reported in a study of laboratory-prepared surfaces using *S. aureus*, although with much higher recovery of 83.7% and 57% for premoistened and dry swabs, respectively.^15^ Another potential differentiating factor between swabs was the medium utilized in collection. The sponge-sticks we selected contained “neutralizing buffer,” which is designed to counteract commonly used disinfectants.^16^ It is possible that this medium resulted in greater viability compared to the liquid Amies medium used for flocked swabs; however, this was not directly compared. Lastly, while it is possible that the greater surface area sampled resulted in greater recovery, this was not borne out in our laboratory-prepared surface experiment, where equal surface areas were sampled.

While we demonstrated the superiority of sponge-sticks for culture-based recovery, we found that flocked swabs resulted in greater recovery of bacterial DNA. This difference was largely attributed to 24 sponge-stick specimens, where there was no quantifiable 16S rRNA gene sequence detected. However, the flocked swab method remained superior even after excluding these results. It is possible that the greater volume associated with processing the sponge-stick specimen resulted in dilution of bacterial DNA that was only incompletely recovered by centrifugation. Inhibition of PCR reactions has been described with cellulose-containing specimens.^17^ The sponge-sticks used in these experiments were primarily composed of cellulose, however, we were unable to confirm if residual cellulose was present after DNA extraction or if PCR inhibition occurred. Additionally, it is possible that despite the stomaching homogenizing process, that bacteria remained adhered to the sponge material and did not extract into solution.

Prior research comparing swabbing modalities for environmental sampling in healthcare settings is limited. This work has mostly focused on the recovery of *Bacillus* species, demonstrating similar performance of swabs to RODAC plates^18^ and improved performance of nylon flocked swabs to cotton swabs.^19^ Goverde et al compared multiple swabbing methods and found that the nylon flocked swab performed best among the 3 swabs examined, with the highest mean recovery of 43.6% compared to 24.1% and 14.1% with other methods. This recovery was higher than that we detected in our study of laboratory-prepared surfaces using flocked swabs. Notably, the nylon flocked swabs used in the Goverde et al study were processed either by direct plating to bacterial growth media or using membrane filtration, methods that we did not utilize due to our use of multiple selective media in parallel. Notably, these studies did not investigate the effectiveness of the recovery of organisms of concern in healthcare epidemiology and focused primarily on laboratory-prepared surfaces. In contrast, Thom et al^20^ investigated recovery of *A. baumannii* within hospital rooms using both a cellulose sponge and cotton swab premoistened with PBS. They found that the flocked swab was superior to the sponge method with a recovery sensitivity of 87% compared to 75%. Our study differed in the use of dry flocked nylon swabs in patient rooms rather than premoistened cotton swabs, and in that, we did not perform a broth enrichment step. Comparison of flocked swabs to sponge-sticks for environmental sampling has been limited in previous research.

Our study had several limitations. Due to concern that repeated sampling of the same surface area would reduce yield in later samples, flocked swabs sampled a smaller surface area compared to sponge-sticks for our hospital room comparison. An alternative strategy would have been to randomize the order of sampling and evaluate the impact of swab order on recovery. While this was not feasible in our experiment, we were able to investigate recovery from a fixed surface area in a laboratory-prepared surface limited to a single organism and single surface type. Additionally, we did not compare the effectiveness of premoistened compared with dry flocked swabs in our real-world comparison, which likely would have resulted in improved performance. Lastly, our study was limited to several commonly used swabs and was not exhaustive of available products.

In conclusion, the optimal swabbing modality depends on the priority of molecular versus culture-based experiments. Our findings suggest that sponge-stick collection is preferred for culture-based modalities in comparison to dry flocked swabs, but that premoistened swabs may result in improved performance. In contrast, flocked swab collection was superior for molecular detection of bacteria. While sponge-stick use was superior for culture-based recovery, it is more labor intensive to process these specimens and requires special equipment not present in many hospital microbiology laboratories. Future studies can be conducted to further optimize sampling, including determining ideal surface areas for bacterial recovery.

## Supporting information

Ziegler et al. supplementary material Ziegler et al. supplementary material

## Supplementary material

The supplementary material for this article can be found at https://doi.org/10.1017/ice.2025.87
