# Supplementary material for: Comparative performance of sponge versus flocked swabs for culture-based and metagenomic detection of microbial contamination in the healthcare environment
Source: Infect Control Hosp Epidemiol. 2025 May 27;46(8):831–6. doi: 10.1017/ice.2025.87 (PMC12483624; doi:10.1017/ice.2025.87)
Supplement: Ziegler et al. supplementary material [file S0899823X2500087Xsup001.docx]

**Supplementary Methods**

*Description of Laboratory Prepared Surface Preparation and Sampling*

Preparation of Surfaces

Using Staphylococcus aureus strain 493NT, two dilutions were created by McFarland optical density and colony counting (Supplementary Figure 1). The dilutions were determined by previous dilutions sets and colony counting to a readable number of colonies to be present on a blood agar plate (BAP) (10 – 100 colonies/ BAP). A 0.5 McFarland of SSA was diluted in saline to an estimated 1 x 10^6^ CFU (Solution 1), and serial to 1 x 10^5^ CFU (Solution 2) and 1 x 10^4^ CFU (Solution 3, control use only). Each control was plated to BAP with three different inoculation volumes (1uL, 10uL, 100uL) to account for colony crowding and very dilute samples.

Comparison of Flocked and Sponge Swab Recovery

100uL of Solution 1 and Solution 2 (1:10 dilution of CFU) were inoculated and spread on to a 130mm x 85mm plastic surface with a 10mm border on each side. A total of 8 surfaces were inoculated. Immediately after inoculation, separate flocked swabs (BD Eswabs) and sponge-sticks (3M Sponge-Stick) swabbed a surface for both solutions surfaces. Surfaces were swabbed by applying the swab to the surface with moderate pressure, covering the surface in an ‘S’ pattern and rotating the surface, and the swab, for a total of four surface passes. Surface areas sampled were identical for all samples. Samples were processed according to protocol described in the methods section “MDRO Culture”. Post processing, the flocked swab was plated to BAP in three different amounts (10uL, 50uL, 100uL). The sponge-stick was plated on to BAP in four different amounts (10uL, 50uL, 100uL, and 1ml). The four remaining inoculated surfaces remained in the biosafety cabinet until completely dried (30 – 35mins at 60% humidity). The remaining surfaces were swabbed and processed similarly to the wet culture swabs. Solution concentrations were determined by colony count of control plates. Colony counts were performed on each of the culture plates and averaged. The average amount of CFU/ml from colony counts was compared to the control concentrations.

Comparison of Dry versus Premoistened Flocked Swab Recovery

100uL of the Solution 1 and Solution 2 (1:10 dilution of CFU) were inoculated and spread on to a 130mm x 85mm plastic surface with a 10mm border on each side. A total of 8 surfaces were inoculated. The pre-moistened flocked swab (BD Eswab) was prepared by dipping the swab into the liquid Amies transport media and removing excess fluid by pushing the swab against the inside of the container. Immediately after inoculation, surfaces were swabbed by applying the swab to the surface with moderate pressure, covering the surface in an ‘S’ pattern and rotating the surface, and the swab, for a total of four surface passes. Surface areas sampled were identical for all samples. Samples were processed according to protocol described in the methods section “MDRO Culture”. Post processing, the dry flocked swabs and pre-moistened flocked swabs were plated on to BAP with three different amounts of transport media (10uL, 50uL, 100uL). The four remaining inoculated surfaces remained in the BSC until completely dried (30 – 35mins with 60% humidity). The remaining surfaces were swabbed and processed similar to the wet culture swabs. Solution concentrations were determined by colony count of control plates. Colony counts were performed on each of the culture plates and averaged. The average amount of CFU/ml from colony counts was compared to the control concentrations.

**Supplementary Tables & Figures**

**Supplementary Table 1. Results of Laboratory Prepared Surface Recovery – Comparing Flocked to Sponge Swab**

| **Swab Type** | **Surface Preparation** | **CFU** | **CFU Recovered^a^** | **Recovery %** | **Average Recovery %** |
| --- | --- | --- | --- | --- | --- |
|  |  | **Control** |  |  |  |
| Sponge | Wet | 8320 | 5673.33 | 68.19 | 59.4 |
|  |  | 909 | 460 | 50.61 |  |
|  | Dry | 8320 | 206.67 | 2.48 | 1.61 |
|  |  | 909 | 6.67 | 0.73 |  |
| Flocked | Wet | 41600 | 3200 | 7.69 | 7.95 |
|  |  | 4550 | 373.33 | 8.21 |  |
|  | Dry | 41600 | 160 | 0.38 | 0.19 |
|  |  | 4550 | 0 | 0 |  |

^a^Averaged readable colony counts from three inoculation volumes (100uL, 50uL, 10uL) to blood agar

**Supplementary Table 2. Results of Laboratory Prepared Surface Recovery – Comparing Premoistened to Dry Flocked Swab**

| **Flocked Swab Preparation** | **Surface Preparation** | **CFU**  **Control** | **CFU Recovered** | **Recovery %** | **Average Recovery %** |
| --- | --- | --- | --- | --- | --- |
| Dry | Wet | 25900 | 3140 | 12.12 | 13.05 |
|  |  | 3600 | 503.33 | 13.98 |  |
|  | Dry | 25900 | 33.33 | 0.13 | 0.07 |
|  |  | 3600 | 0 | 0 |  |
| Premoistened | Wet | 25900 | 9960 | 38.46 | 33.86 |
|  |  | 3600 | 1053.33 | 29.26 |  |
|  | Dry | 25900 | 300 | 1.16 | 1.37 |
|  |  | 3600 | 56.67 | 1.57 |  |

^a^Averaged readable colony counts from three inoculation volumes (100uL, 50uL, 10uL) to blood agar

**Supplementary Figure 1. Overview of the serial dilutions and inoculum amounts for preparation of surfaces**

**
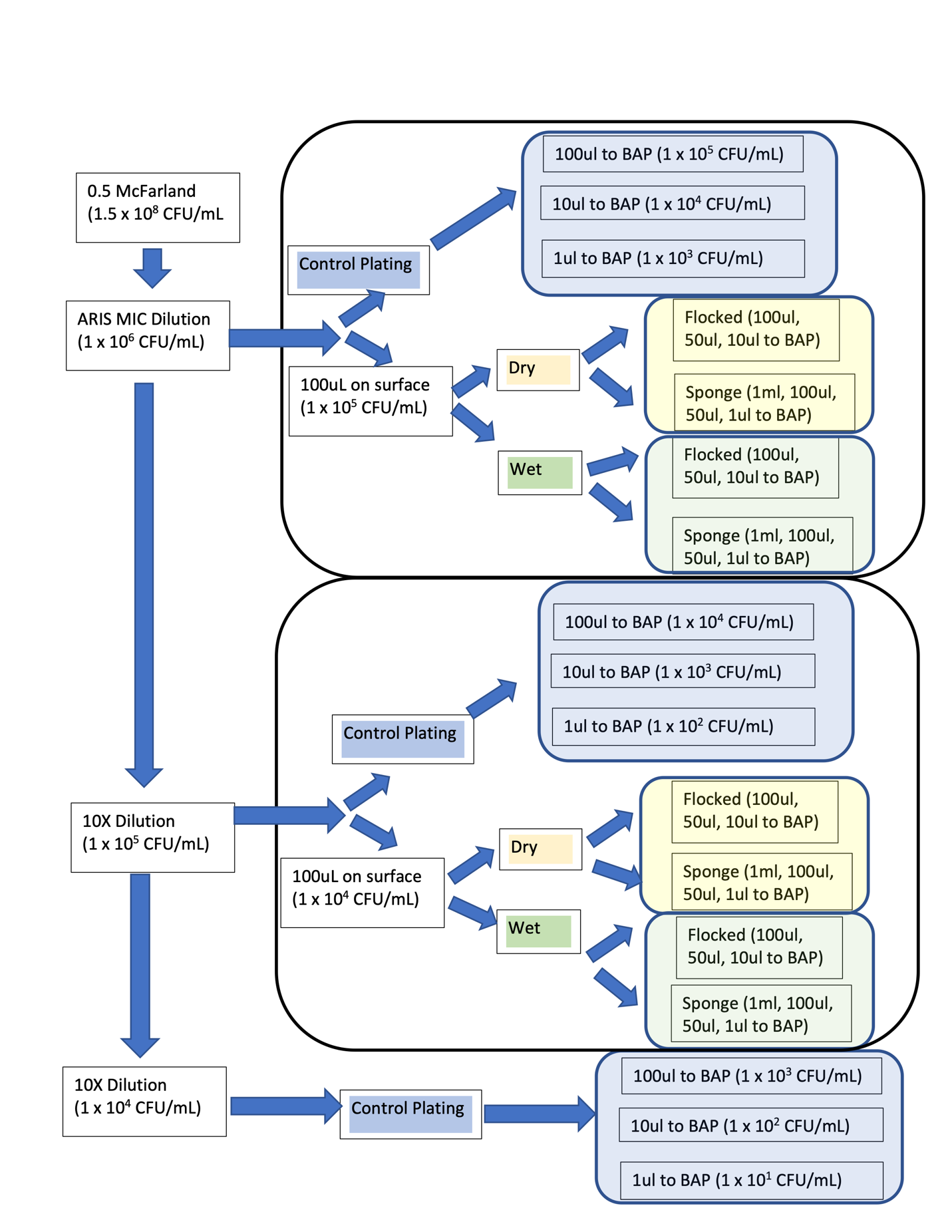
**
